# Synthesis and structure of ethyl 2-[(4-oxo-3-phenyl-3,4-di­hydro­quinazolin-2-yl)sulfan­yl]acetate

**DOI:** 10.1107/S2056989020005071

**Published:** 2020-04-17

**Authors:** Cong Nguyen Tien, Quang Nguyen Tan, Dung Pham Duc, Phuong Tran Hoang, Dat Nguyen Dang, Luong Truong Minh, Luc Van Meervelt

**Affiliations:** a Faculty of Chemistry, Ho Chi Minh City University of Education, 280 An Duong Vuong Street, Ho Chi Minh City, 700000, Vietnam; b Phan Boi Chau High School, 70 Le Hong Phong Street, Binh Thuan Province, 77000, Vietnam; c Faculty of Chemistry, University of Science, 227 Nguyen Van Cu Street, Ho Chi Minh City, 721337, Vietnam; d Faculty of Chemistry, Hanoi National University of Education, 136 Xuan Thuy, Cau Giay, Hanoi, Vietnam; e Department of Chemistry, KU Leuven, Biomolecular Architecture, Celestijnenlaan 200F, Leuven (Heverlee), B-3001, Belgium

**Keywords:** crystal structure, quinazolin-4-one, hydrogen bonding, Hirshfeld analysis

## Abstract

The title compound, C_18_H_16_N_2_O_3_S, was synthesized by reaction of 2-mercapto-3-phenyl­quinazolin-4(3*H*)-one with ethyl chloro­acetate. The quinazoline ring forms a dihedral angle of 86.83 (5)° with the phenyl ring. The terminal methyl group is disordered by a rotation of about 60° in a 0.531 (13): 0.469 (13) ratio. In the crystal, C—H⋯O hydrogen-bonding inter­actions result in the formation of columns running in the [010] direction. Two parallel columns further inter­act by C—H⋯O hydrogen bonds. The most important contributions to the surface contacts are from H⋯H (48.4%), C⋯H/H⋯C (21.5%) and O⋯H/H⋯O (18.7%) inter­actions, as concluded from a Hirshfeld analysis.

## Abstract 2

In the title compound, C_18_H_16_N_2_O_3_S, the dihedral angle between the mean planes of the quinazoline and phenyl rings is 86.83 (5)°. In the crystal, C—H⋯O inter­actions link the mol­ecules into infinite columns along the *b*-axis direction. Parallel columns inter­act by additional C—H⋯O hydrogen bonds.

## Chemical context   

Hybrid derivatives, where quinazolin-4-one is incorporated with different heterocycles, possess a variety of biological effects including anti­cancer (Khalil *et al.*, 2003 ▸; Gursoy & Karal, 2003 ▸; Gawad *et al.*, 2010 ▸; Elfekki *et al.*, 2014 ▸; Alanazi *et al.*, 2016 ▸; El-Sayed *et al.*, 2017 ▸; Nguyen *et al.*, 2019 ▸), anti­convulsant (El-Azab *et al.*, 2013 ▸) and anti­microbial (Pandey *et al.*, 2009 ▸; Al-Khuzaie & Al-Majidi, 2014 ▸; Al-Majidi & Al-Khuzaie, 2015 ▸; Lv *et al.*, 2018 ▸; Godhani *et al.*, 2016 ▸) activities. Some derivatives of 2-mercapto-3-(4-meth­oxy­phen­yl)quin­azo­lin-4(3*H*)-one containing the thia­zolidine-4-one moiety have been found to have good anti­tuberculosis activity (Godhani *et al.*, 2016 ▸). In addition, many amide and *N*-substituted hydrazide compounds derived from 2-mercapto-3-phenyl­quinazolin-4-one have been demonstrated to have valuable biological activities such as anti­tumor (Al-Suwaidan *et al.*, 2016 ▸, 2017 ▸; Mohamed *et al.*, 2016 ▸), anti­convulsant (El-Helby & Wahab, 2003 ▸) and anti­bacterial (Lfta *et al.*, 2016 ▸) activity. The capacity to increase the HDL cholesterol activity of some *N*-substituted compounds containing a quinazolin-4-one moiety has also been investigated (Deshmukh & Dhongade, 2004 ▸).
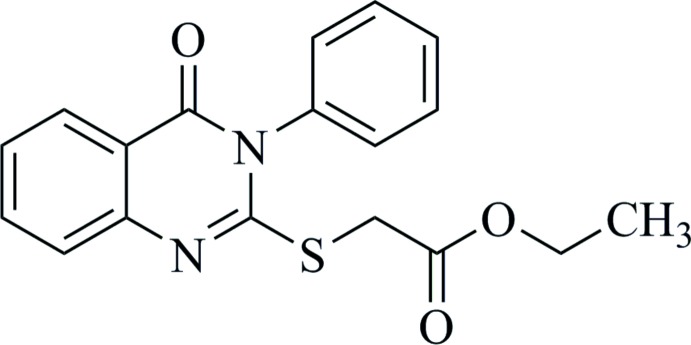


Ethyl 2-[(4-oxo-3-phenyl-3,4-di­hydro­quinazolin-2-yl)sulf­an­yl]­acetate is an inter­mediate compound in the synthesis process of both *N*-substituted and heterocyclic compounds containing a quinazolin-4-one moiety. The synthesis and properties of ethyl 2-[(4-oxo-3-phenyl-3,4-di­hydro­quinazolin-2-yl)thio]­acetate have therefore attracted much attention.

**Figure 1:**
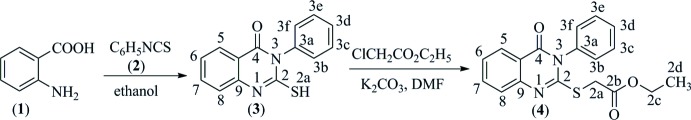
Reaction scheme for the synthesis of the title compound (**4**).

As shown in Fig. 1 ▸, 2-mercapto-3-phenyl­quinazolin-4(3*H*)-one (**3**) was obtained by the reaction of anthranilic acid (**1**) and phenyl iso­thio­cyanate (**2**) (Nguyen *et al.*, 2019 ▸). The IR spectrum of (**3**) shows the stretching vibrations of N—H (3217 and 3134 cm^−1^) and C=O (1659 cm^−1^) bonds, indicating that (**3**) exists in the thione form (Al-Majidi & Al-Khuzaie, 2015 ▸). In the ^1^H NMR spectrum, besides signals of nine protons in the aromatic area, there is a singlet signal with the intensity of 1H at δ 13.05 ppm attributed to the proton of the thiol group. In an alkaline medium, (**3**) exists in the thiol­ate form and reacts easily with ethyl chloro­acetate to yield (**4**). In the IR spectrum of (**4**), the disappearance of the NH stretching and the presence of a strong C=O absorption at 1732 cm^−1^ indicate the existence of an ester compound. In the ^1^H NMR spectrum of (**4**), the signal at δ 13.05 ppm disappears and three new signals in the aliphatic area [*singlet* signal at δ 3.99 (2H), *quartet* signal at δ 4.15 (2H) and *triplet* signal at δ 1.23 ppm (3H)] are consistent with the presence of the –CH_2_COOCH_2_CH_3_ moiety in (**4**).

As no X-ray crystallographic information is available for this ester, we have determined the crystal structure by single-crystal X-ray diffraction and a Hirshfeld surface analysis has been performed to gain further insight into the inter­molecular inter­actions.

## Structural commentary   

**Figure 2:**
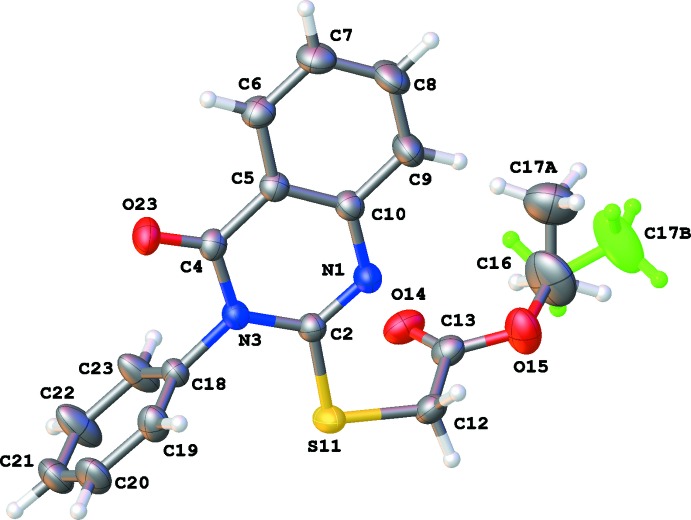
The mol­ecular structure of the title compound, showing the atom-labelling scheme and displacement ellipsoids at the 50% probability level. Methyl group C17*B* [occupancy 0.469 (13)] is shown in green.

The title compound crystallizes in the space group *P*2_1_/*n* with four mol­ecules in the unit cell. The asymmetric unit of the title compound is illustrated in Fig. 2 ▸. The C17 methyl group is disordered over two orientations by a rotation of about 60° about the O15—C16 bond in a 0.531 (13): 0.469 (13) ratio. The quinazoline ring system is almost planar (r.m.s. deviation = 0.0207 Å). The angle between the two fused six-membered rings is 2.05 (9)°. The substituents S11, C18 and O23 deviating by −0.0951 (17), −0.140 (2) and 0.108 (2) Å, respectively, from the best plane through the quinazoline ring system. This plane makes an angle of 86.83 (5)° with the plane of the C18–C23 phenyl ring (r.m.s. deviation = 0.0052 Å). The dihedral angle between the best planes through the acetate atoms (C12, C13, O14 and O15) and the quinazoline ring system is 75.21 (5)°. A short intra­molecular C16—H16*B*⋯O14 contact is observed [C16—H16*B* = 0.97 Å, H16*B*⋯O14 = 2.28 Å, C16⋯O14 = 2.655 (4) Å, C16—H16*B*⋯O14 = 102°].

**Figure 3:**
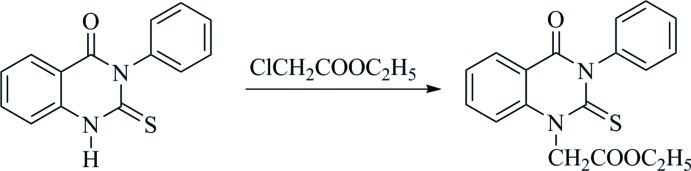
Reaction scheme for the thione tautomer of (**3**) with ethyl chloro­acetate resulting in ethyl 2-(4-oxo-3-phenyl-2-thioxo-3,4-di­hydro­quinazolin-1(2*H*)-yl)acetate as reaction product.

Theoretically, compound (**3**) may exist in the thione form, namely 3-phenyl-2-thioxo-2,3-di­hydro­quinazolin-4(1*H*)-one. Therefore, it could react with ethyl chloro­acetate to give ethyl 2-(4-oxo-3-phenyl-2-thioxo-3,4-di­hydro­quinazolin-1(2*H*)-yl)acetate as illustrated in Fig. 3 ▸. However, our current structure determination indicates that the final product is ethyl 2-[(4-oxo-3-phenyl-3,4-di­hydro­quinazolin-2-yl)sulfanyl]­acetate (**4**), which proves that in the alkaline environment, (**3**) converts into the thiol­ate form and then reacts with ethyl chloro­acetate to yield the title compound (**4**).

## Supra­molecular features and Hirshfeld surface analysis   

**Figure 4:**
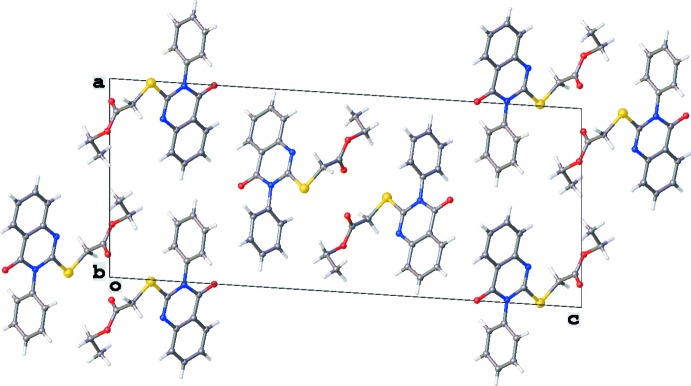
View of the crystal packing of the title compound along the [010] direction. Only the major component of the disordered C17 methyl group is shown.

The crystal packing is mainly characterized by C—H⋯O hydrogen-bonding inter­actions (Table 1 ▸, Figs. 4 ▸ and 5 ▸). Columns running in the [010] direction are formed by C12—H12*B*⋯O14^ii^ and C19—H19⋯O23^ii^ inter­actions, which results also in a short S11⋯H23^ii^ contact of 3.020 Å [symmetry code: (ii) *x*, *y* + 1, *z*]. Two parallel columns inter­act *via* C7—H7⋯O23^i^ hydrogen-bonding inter­actions [symmetry code: (i) −*x* + 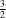, *y* − 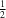, −*z* + 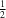]. No voids, C—H⋯π inter­actions or π–π stackings are observed in the crystal packing.

**Figure 6:**
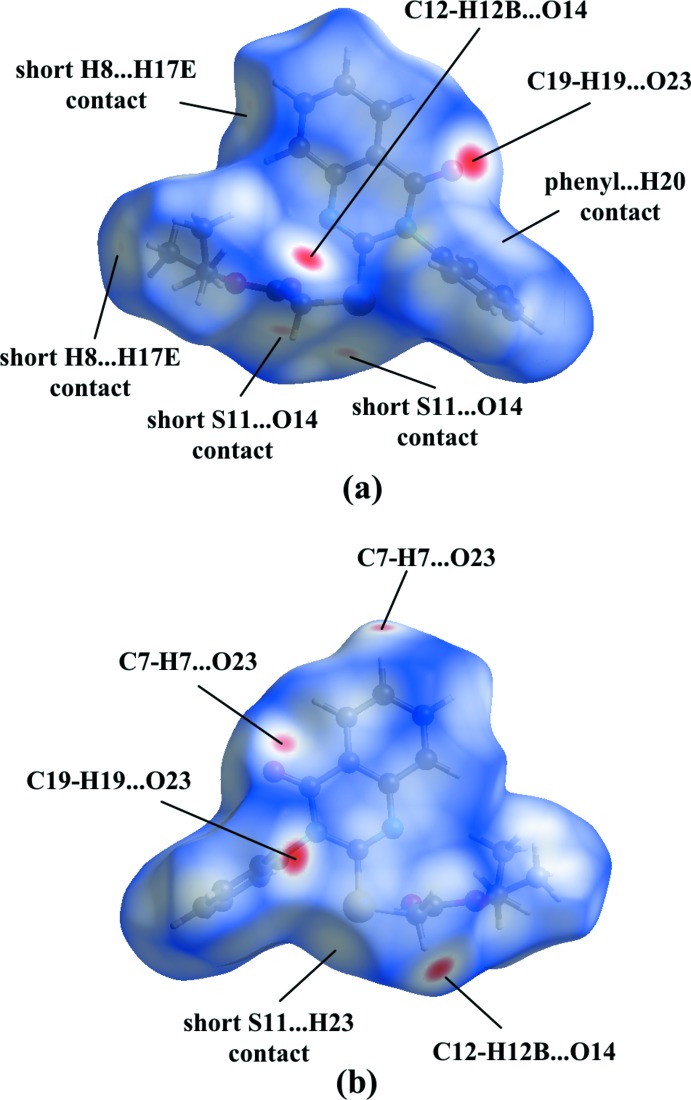
The Hirshfeld surface of (**4**) mapped over *d*_norm_ for the title compound in the range −0.2419 to 1.2857 a.u.

**Figure 7:**
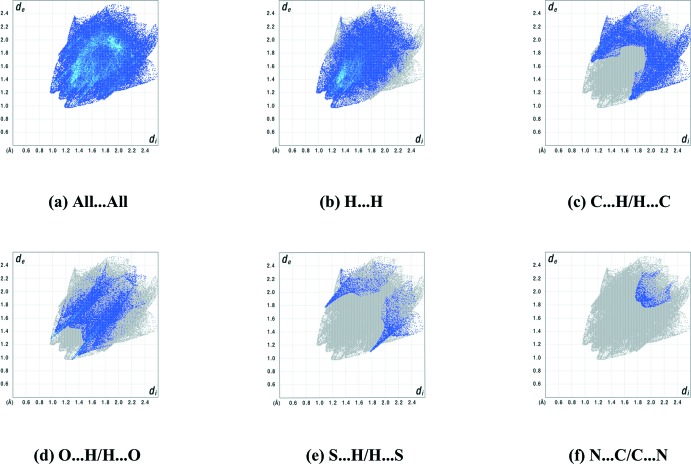
Full two-dimensional fingerprint plots for the title compound, showing (*a*) all inter­actions, and delineated into (*b*) H⋯H, (*c*) C⋯H/H⋯C, (*d*) O⋯H/H⋯O, (*e*) S⋯H/H⋯S and (*f*) N⋯C/C⋯N inter­actions. The *d*_i_ and *d*_e_ values are the closest inter­nal and external distances (in Å) from a given point on the Hirshfeld surface.

In order to gain further insight into the inter­molecular inter­actions, a Hirshfeld surface and two-dimensional fingerprint plots were calculated using *CrystalExplorer* (Turner *et al.*, 2017 ▸). The Hirshfeld surface mapped over *d*_norm_ (Fig. 6 ▸) shows the expected bright-red spots near atoms O14, O23, H7, H12*B* and H19 involved in the C—H⋯O hydrogen-bonding inter­actions described above. In addition, the faint-red spots near atoms S11 and O14 indicate a short S⋯O contact [3.2128 (16) Å]. Small faint-red spots appear near atoms H8 and H17*E* are due to a short H8⋯H17*E* contact (2.352 Å). The S11⋯H23 contact mentioned is only visible as a white spot, while a white region above the C18–C23 phenyl ring is present because of the proximity of atom H20. The distance between H20 and the centroid of this phenyl ring of 3.204 Å, however, is too long for a C—H⋯π inter­action. The fingerprint plots (Fig. 7 ▸) illustrate that the largest contributions to the Hirshfeld surface come from H⋯H contacts (48.4%), followed by significant contributions by reciprocal C⋯H/H⋯C (21.5%) and O⋯H/H⋯O (18.7%) contacts. Smaller contributions are from S⋯H/H⋯S (4.0%), N⋯C/C⋯N (1.6%), C⋯C (1.6%), C⋯S/S⋯C (1.4%), N⋯H/H⋯N (1.3%), S⋯O/O⋯S (1.0%), N⋯S/S⋯N (1.0%) and O⋯O contacts (0.1%).

## Database survey   

A search of the Cambridge Structural Database (CSD, Version 5.41, update of November 2019; Groom *et al.*, 2016 ▸) for 4-oxo-3,4-di­hydro­quinazoline gave 645 hits, of which 141 have a phenyl group at position N3 and 27 have a sulfur atom at position C2. A combination of both substitutions (without a link between the two) results in a set of 10 hits, which was used for further analysis. The dihedral angle between the least-squares planes through the quinazoline and phenyl rings varies between 71.99° (CSD refcode MUDGID; Saeed *et al.*, 2014 ▸) and 86.46° (CSD refcode GUWDIM; Rimaz *et al.*, 2009 ▸) with an average of 81.63°. The dihedral angle does not depend on eventual *ortho* subsitution of the phenyl ring, as illustrated by the structures MUDGID (71.99°) and MUDNAC (85.90°; Saeed *et al.*, 2014 ▸), which both have an *o*-toluidine substituent at position N3. The almost perpendicular mutual orientation of both rings is also observed for the title compound.

## Synthesis and crystallization   

Anthranilic acid, phenyl iso­thio­cyanate and ethyl chloro­acetate were purchased from Acros and used without purification. Melting points were measured in open capillary tubes on a Gallenkamp melting point apparatus. IR spectra (ν, cm^−1^) were recorded on FTIR-8400S-SHIMADZU spectrometer using KBr pellets. The NMR spectra were recorded on a Bruker Avance III spectrometer (500 MHz for ^1^H NMR) using residual solvent DMSO-*d*_6_ signals as inter­nal reference. The spin–spin coupling constants (*J*) are given in Hz. Peak multiplicity is reported as *s* (singlet), *d* (doublet), *dd* (doublet-doublet), *t* (triplet), *q* (quartet), *m* (multiplet). The synthetic protocol for title compound (**4**) is shown in Fig. 1 ▸ (Nguyen *et al.*, 2019 ▸).

***Synthesis of 2-mercapto-3-phenyl­quinazolin-4-one (3):***

Phenyl iso­thio­cyanate (**2**) (0.1 mol) was added to the solution of anthranilic acid (**1**) (0.1 mol) and tri­ethyl­amine (3.0 mL) in absolute ethanol (200 mL). The reaction mixture then was refluxed for 4 h. After cooling to room temperature, the reaction mixture was poured into cold water. The resulting solid was filtered and recrystallized from a mixture of DMF and water, then washed with cold ethanol to give the product (**3**). M.p. 569 K; yield 80%. IR (KBr, cm^−1^): 3217, 3134 (N—H), 3028 (C—H aromatic), 1659 (C=O), 1618, 1524, 1485 (C=N, C=C aromatic). ^1^H NMR [Bruker XL-500, 500 MHz, *d*_6_-DMSO, *δ* (ppm), *J* (Hz)]: 13.05 (*s*, 1H, H^2a^), 7.96 (*d*, 1H, ^3^ *J* = 8.0 Hz, H^5^), 7.80 (*dd*, 1H, ^3^ *J*_1_ = ^3^ *J*_2_ = 8.0 Hz, H^7^), 7.50–7.40 (*m*, 3H, H^8,3c,3e^), 7.42 (*dd*, 1H, ^3^ *J*_1_ = ^3^ *J*_2_ = 7.5 Hz, H^6^), 7.36 (*dd*, 1H, ^3^ *J*_1_ = ^3^ *J*_2_ = 7.5 Hz, H^3d^), 7.29 (*d*, 2H, ^3^ *J* = 7.5 Hz, H^3b,3f^).

***Synthesis of ethyl 2-[(4-oxo-3-phenyl-3,4-di­hydro­quinazolin-2-yl) sulfanyl]­acetate (4):***

A mixture of (**3**) (20 mmol) and anhydrous potassium carbonate (20 mmol) in dry DMF (30 mL) was stirred for 30 min, ethyl chloro­acetate (20 mmol) was then added. After refluxing for 5 h, the reaction mixture was cooled to room temperature and poured into ice-cold water. The white precipitate was filtered off and recrystallized from ethanol to afford crystals of (**4**). Colourless crystals, m.p. 485 K, yield 65%. IR (KBr, cm^−1^): 3059 (C—H aromatic), 2976, 2906 (C—H aliphatic), 1732 (C=O ester), 1680 (C=O ketone), 1607, 1598, 1468 (C=N, C=C aromatic). ^1^H NMR [Bruker XL-500, 500 MHz, *d*_6_-DMSO, *δ* (ppm), *J* (Hz)]: 8.09 (*d*, 1H, ^3^ *J* = 8.0 Hz, H^5^), 7.84 (*d*, 1H, ^3^ *J* = 7.5 Hz, H^8^), 7.61-7.48 (*m*, 7H, H^6,7,3b,3c,3d,3e,3f^), 4.15 (*q*, 2H, ^3^ *J* = 7.0 Hz, H^2c^), 3.99 (*s*, 2H, H^2a^), 1.23 (*t*, 3H, ^3^ *J* = 7.0 Hz, H^2d^).

## Refinement   

Crystal data, data collection and structure refinement details are summarized in Table 2 ▸. The methyl group C17 is disordered over two positions with population parameters 0.531 (13) and 0.469 (13)]. The H atoms were placed in idealized positions and included as riding contributions with *U*_iso_(H) values of 1.2*U*_eq_ or 1.5*U*_eq_ of the parent atoms, with C—H distances of 0.93 (aromatic), 0.97 (CH_2_) and 0.96 Å (CH_3_). In the final cycles of refinement, two outliers were omitted.

## Supplementary Material

Crystal structure: contains datablock(s) I. DOI: 10.1107/S2056989020005071/dj2002sup1.cif

Structure factors: contains datablock(s) I. DOI: 10.1107/S2056989020005071/dj2002Isup2.hkl

Supporting information file. DOI: 10.1107/S2056989020005071/dj2002Isup3.cml

CCDC reference: 1996127

Additional supporting information:  crystallographic information; 3D view; checkCIF report

## Figures and Tables

**Figure 5:**
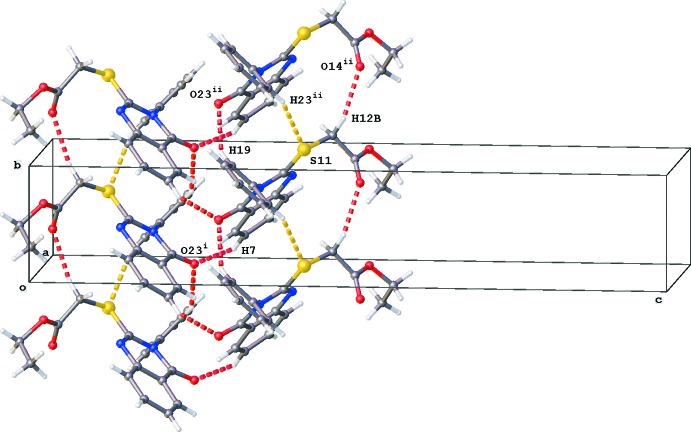
Partial crystal packing of the title compound showing two parallel columns running in the [010] direction. Inter­molecular C—H⋯O inter­actions are shown as red dashed lines (see Table 1 ▸ for details), C—H⋯S inter­actions as yellow dashed lines. Only the major component of the disordered C17 methyl group is shown.

**Table 1:** Hydrogen-bond geometry (Å, °)

*D*—H⋯*A*	*D*—H	H⋯*A*	*D*⋯*A*	*D*—H⋯*A*
C7—H7⋯O23^i^	0.93	2.59	3.452 (3)	155
C12—H12*B*⋯O14^ii^	0.97	2.42	3.311 (3)	153
C19—H19⋯O23^ii^	0.93	2.41	3.236 (2)	148

Symmetry codes: (i) 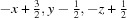; (ii) 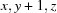.

**Table 2:** Experimental details

Crystal data	
Chemical formula	C_18_H_16_N_2_O_3_S
*M*_r_	340.39
Crystal system, space group	Monoclinic, *P*2_1_/*n*
Temperature (K)	293
*a*, *b*, *c* (Å)	11.8865 (6), 5.1298 (3), 28.2942 (14)
β (°)	93.667 (4)
*V* (Å^3^)	1721.72 (16)
*Z*	4
Radiation type	Mo *K*α
μ (mm^−1^)	0.21
Crystal size (mm)	0.5 × 0.15 × 0.15

Data collection	
Diffractometer	Rigaku Oxford Diffraction SuperNova, single source at offset/far, Eos
Absorption correction	Multi-scan (*CrysAlis PRO*; Rigaku OD, 2018 ▸)
*T*_min_, *T*_max_	0.715, 1.000
No. of measured, independent and observed [*I* > 2σ(*I*)] reflections	18522, 3533, 2875
*R*_int_	0.024
(sin θ/λ)_max_ (Å^−1^)	0.625

Refinement	
*R*[*F* ^2^ > 2σ(*F* ^2^)], *wR*(*F* ^2^), *S*	0.043, 0.111, 1.08
No. of reflections	3533
No. of parameters	229
H-atom treatment	H-atom parameters constrained
Δρ_max_, Δρ_min_ (e Å^−3^)	0.15, −0.22

Computer programs: *CrysAlis PRO* (Rigaku OD, 2018 ▸), *SHELXT* (Sheldrick, 2015*a* ▸), *SHELXL* (Sheldrick, 2015*b* ▸) and *OLEX2* (Dolomanov *et al.*, 2009 ▸).

## supplementary crystallographic information

### Crystal data

**Table:**

C_18_H_16_N_2_O_3_S	*F*(000) = 712
*M**_r_* = 340.39	*D*_x_ = 1.313 Mg m^−^^3^
Monoclinic, *P*2_1_/*n*	Mo *K*α radiation, λ = 0.71073 Å
*a* = 11.8865 (6) Å	Cell parameters from 7343 reflections
*b* = 5.1298 (3) Å	θ = 2.9–26.9°
*c* = 28.2942 (14) Å	µ = 0.21 mm^−^^1^
β = 93.667 (4)°	*T* = 293 K
*V* = 1721.72 (16) Å^3^	Needle, colourless
*Z* = 4	0.5 × 0.15 × 0.15 mm

### Data collection

**Table:**

Rigaku Oxford Diffraction SuperNova, Single source at offset/far, Eos diffractometer	3533 independent reflections
Radiation source: micro-focus sealed X-ray tube, SuperNova (Mo) X-ray Source	2875 reflections with *I* > 2σ(*I*)
Mirror monochromator	*R*_int_ = 0.024
Detector resolution: 15.9631 pixels mm^-1^	θ_max_ = 26.4°, θ_min_ = 2.7°
ω scans	*h* = −14→14
Absorption correction: multi-scan (CrysAlisPro; Rigaku OD, 2018)	*k* = −6→6
*T*_min_ = 0.715, *T*_max_ = 1.000	*l* = −35→35
18522 measured reflections	

### Refinement

**Table:**

Refinement on *F*^2^	Primary atom site location: dual
Least-squares matrix: full	Hydrogen site location: inferred from neighbouring sites
*R*[*F*^2^ > 2σ(*F*^2^)] = 0.043	H-atom parameters constrained
*wR*(*F*^2^) = 0.111	*w* = 1/[σ^2^(*F*_o_^2^) + (0.0409*P*)^2^ + 0.5306*P*] where *P* = (*F*_o_^2^ + 2*F*_c_^2^)/3
*S* = 1.08	(Δ/σ)_max_ < 0.001
3533 reflections	Δρ_max_ = 0.14 e Å^−^^3^
229 parameters	Δρ_min_ = −0.22 e Å^−^^3^
0 restraints	

### Special details

**Table:**

Geometry. All esds (except the esd in the dihedral angle between two l.s. planes) are estimated using the full covariance matrix. The cell esds are taken into account individually in the estimation of esds in distances, angles and torsion angles; correlations between esds in cell parameters are only used when they are defined by crystal symmetry. An approximate (isotropic) treatment of cell esds is used for estimating esds involving l.s. planes.

### Fractional atomic coordinates and isotropic or equivalent isotropic displacement parameters (Å^2^)

**Table:**

	*x*	*y*	*z*	*U*_iso_*/*U*_eq_	Occ. (<1)
N1	0.68674 (11)	0.7908 (3)	0.38689 (5)	0.0521 (4)	
C2	0.58088 (13)	0.8340 (3)	0.37790 (6)	0.0453 (4)	
N3	0.51456 (10)	0.7160 (3)	0.34201 (5)	0.0438 (3)	
C4	0.55985 (14)	0.5351 (3)	0.31132 (6)	0.0456 (4)	
C5	0.67846 (13)	0.4761 (3)	0.32214 (6)	0.0450 (4)	
C6	0.73246 (16)	0.2867 (4)	0.29607 (7)	0.0577 (5)	
H6	0.692325	0.197199	0.271885	0.069*	
C7	0.84377 (17)	0.2334 (4)	0.30609 (8)	0.0702 (6)	
H7	0.879740	0.107617	0.288799	0.084*	
C8	0.90293 (17)	0.3672 (5)	0.34210 (9)	0.0794 (7)	
H8	0.979020	0.331142	0.348609	0.095*	
C9	0.85182 (16)	0.5518 (5)	0.36837 (8)	0.0709 (6)	
H9	0.893080	0.639601	0.392451	0.085*	
C10	0.73754 (14)	0.6078 (4)	0.35893 (6)	0.0489 (4)	
S11	0.50921 (4)	1.05531 (10)	0.41245 (2)	0.06194 (18)	
C12	0.62397 (16)	1.1534 (4)	0.45253 (7)	0.0564 (5)	
H12A	0.686170	1.209133	0.434376	0.068*	
H12B	0.600486	1.301892	0.470701	0.068*	
C13	0.66465 (17)	0.9421 (4)	0.48617 (7)	0.0563 (5)	
O14	0.61472 (13)	0.7513 (3)	0.49620 (5)	0.0728 (4)	
O15	0.76681 (14)	1.0046 (3)	0.50483 (6)	0.0907 (5)	
C16	0.8156 (3)	0.8266 (9)	0.54064 (14)	0.1386 (14)	
H16A	0.856216	0.924744	0.565576	0.166*	0.531 (13)
H16B	0.756086	0.729319	0.554614	0.166*	0.531 (13)
H16C	0.780435	0.857679	0.570144	0.166*	0.469 (13)
H16D	0.798109	0.649159	0.530875	0.166*	0.469 (13)
C17A	0.8872 (9)	0.6589 (19)	0.5203 (4)	0.129 (4)	0.531 (13)
H17A	0.844520	0.538073	0.500469	0.194*	0.531 (13)
H17B	0.930264	0.565109	0.544649	0.194*	0.531 (13)
H17C	0.937310	0.755551	0.501607	0.194*	0.531 (13)
C17B	0.9293 (6)	0.850 (3)	0.5485 (4)	0.152 (7)	0.469 (13)
H17D	0.964924	0.820026	0.519528	0.228*	0.469 (13)
H17E	0.955908	0.724381	0.571772	0.228*	0.469 (13)
H17F	0.947221	1.022473	0.559888	0.228*	0.469 (13)
C18	0.39382 (13)	0.7640 (3)	0.33627 (6)	0.0454 (4)	
C19	0.35129 (17)	0.9418 (4)	0.30424 (7)	0.0620 (5)	
H19	0.399266	1.040013	0.286589	0.074*	
C20	0.23496 (18)	0.9749 (5)	0.29822 (9)	0.0763 (6)	
H20	0.205352	1.096917	0.276528	0.092*	
C21	0.16481 (17)	0.8324 (5)	0.32340 (9)	0.0773 (6)	
H21	0.087168	0.852870	0.318649	0.093*	
C22	0.20844 (17)	0.6590 (6)	0.35575 (11)	0.0981 (9)	
H22	0.160210	0.563733	0.373781	0.118*	
O23	0.50149 (10)	0.4422 (3)	0.27855 (5)	0.0628 (4)	
C23	0.32353 (16)	0.6215 (5)	0.36235 (9)	0.0804 (7)	
H23	0.352638	0.500489	0.384337	0.096*	

### Atomic displacement parameters (Å^2^)

**Table:**

	*U*^11^	*U*^22^	*U*^33^	*U*^12^	*U*^13^	*U*^23^
N1	0.0442 (8)	0.0581 (9)	0.0531 (9)	0.0025 (7)	−0.0027 (6)	−0.0094 (7)
C2	0.0450 (9)	0.0470 (9)	0.0436 (9)	0.0019 (7)	0.0000 (7)	−0.0009 (7)
N3	0.0390 (7)	0.0495 (8)	0.0429 (7)	−0.0008 (6)	0.0014 (6)	−0.0007 (6)
C4	0.0448 (9)	0.0500 (10)	0.0426 (9)	−0.0079 (7)	0.0059 (7)	−0.0003 (7)
C5	0.0436 (8)	0.0490 (9)	0.0432 (9)	−0.0020 (7)	0.0087 (7)	0.0011 (7)
C6	0.0592 (11)	0.0621 (12)	0.0530 (10)	0.0015 (9)	0.0133 (9)	−0.0057 (9)
C7	0.0640 (12)	0.0763 (14)	0.0722 (14)	0.0156 (11)	0.0198 (10)	−0.0067 (11)
C8	0.0448 (10)	0.1025 (18)	0.0911 (16)	0.0192 (11)	0.0054 (10)	−0.0125 (14)
C9	0.0454 (10)	0.0877 (15)	0.0784 (14)	0.0082 (10)	−0.0050 (9)	−0.0172 (12)
C10	0.0415 (9)	0.0551 (10)	0.0503 (10)	0.0012 (8)	0.0051 (7)	−0.0006 (8)
S11	0.0562 (3)	0.0648 (3)	0.0638 (3)	0.0166 (2)	−0.0040 (2)	−0.0173 (2)
C12	0.0662 (11)	0.0442 (10)	0.0583 (11)	0.0019 (9)	−0.0007 (9)	−0.0083 (8)
C13	0.0682 (12)	0.0521 (11)	0.0486 (10)	0.0027 (9)	0.0039 (9)	−0.0064 (9)
O14	0.0954 (11)	0.0510 (8)	0.0736 (10)	−0.0013 (8)	0.0189 (8)	−0.0009 (7)
O15	0.0849 (11)	0.0986 (12)	0.0845 (11)	−0.0084 (9)	−0.0259 (9)	0.0233 (10)
C16	0.130 (3)	0.164 (3)	0.115 (3)	0.017 (3)	−0.037 (2)	0.058 (3)
C17A	0.131 (7)	0.112 (6)	0.143 (7)	0.033 (5)	0.006 (5)	0.020 (5)
C17B	0.093 (5)	0.221 (15)	0.137 (9)	0.000 (6)	−0.030 (5)	0.083 (10)
C18	0.0390 (8)	0.0472 (9)	0.0495 (9)	0.0018 (7)	−0.0019 (7)	0.0005 (7)
C19	0.0591 (11)	0.0629 (12)	0.0625 (12)	−0.0047 (9)	−0.0084 (9)	0.0135 (10)
C20	0.0663 (13)	0.0759 (14)	0.0830 (15)	0.0141 (11)	−0.0229 (12)	0.0171 (12)
C21	0.0445 (10)	0.0862 (16)	0.0997 (17)	0.0125 (11)	−0.0074 (11)	0.0044 (14)
C22	0.0431 (11)	0.113 (2)	0.139 (2)	0.0074 (12)	0.0148 (13)	0.0576 (19)
O23	0.0525 (7)	0.0796 (9)	0.0558 (8)	−0.0115 (7)	0.0002 (6)	−0.0174 (7)
C23	0.0441 (10)	0.0888 (16)	0.1085 (18)	0.0097 (10)	0.0063 (11)	0.0494 (14)

### Geometric parameters (Å, º)

**Table:**

N1—C2	1.287 (2)	S11—C12	1.7896 (19)
N1—C10	1.390 (2)	C12—H12A	0.9700
C17Aa—H17A	0.9600	C12—H12B	0.9700
C17Aa—H17B	0.9600	C12—C13	1.502 (3)
C17Aa—H17C	0.9600	C13—O14	1.188 (2)
C17Bb—H17D	0.9600	C13—O15	1.332 (2)
C17Bb—H17E	0.9600	O15—C16	1.456 (3)
C17Bb—H17F	0.9600	C16—H16A	0.9700
C2—N3	1.384 (2)	C16—H16B	0.9700
C2—S11	1.7541 (17)	C16—H16C	0.9700
N3—C4	1.402 (2)	C16—H16D	0.9700
N3—C18	1.4550 (19)	C16—C17A	1.363 (8)
C4—C5	1.455 (2)	C16—C17B	1.361 (9)
C4—O23	1.219 (2)	C18—C19	1.360 (2)
C5—C6	1.400 (2)	C18—C23	1.362 (3)
C5—C10	1.393 (2)	C19—H19	0.9300
C6—H6	0.9300	C19—C20	1.393 (3)
C6—C7	1.363 (3)	C20—H20	0.9300
C7—H7	0.9300	C20—C21	1.346 (3)
C7—C8	1.383 (3)	C21—H21	0.9300
C8—H8	0.9300	C21—C22	1.356 (3)
C8—C9	1.370 (3)	C22—H22	0.9300
C9—H9	0.9300	C22—C23	1.382 (3)
C9—C10	1.397 (2)	C23—H23	0.9300
			
C2—N1—C10	117.28 (15)	H16Aa—C16—H16B	108.2
N1—C2—N3	124.99 (15)	C17Bb—C16—H16C	108.8
N1—C2—S11	120.31 (13)	C17Bb—C16—H16D	108.8
H17Aa—C17Aa—H17B	109.5	H16Cb—C16—H16D	107.7
H17Aa—C17Aa—H17C	109.5	H12A—C12—H12B	107.7
N3—C2—S11	114.70 (11)	C13—C12—S11	113.59 (13)
C2—N3—C4	121.38 (13)	C13—C12—H12A	108.8
C2—N3—C18	121.27 (13)	C13—C12—H12B	108.8
C4—N3—C18	117.26 (13)	O14—C13—C12	126.89 (19)
N3—C4—C5	114.37 (14)	O14—C13—O15	124.07 (19)
O23—C4—N3	120.51 (15)	O15—C13—C12	109.01 (17)
H17Ba—C17Aa—H17C	109.5	C13—O15—C16	115.9 (2)
H17Db—C17Bb—H17E	109.5	O15—C16—H16A	109.8
H17Db—C17Bb—H17F	109.5	O15—C16—H16B	109.8
H17Eb—C17Bb—H17F	109.5	O15—C16—H16C	108.8
O23—C4—C5	125.12 (16)	O15—C16—H16D	108.8
C6—C5—C4	120.27 (16)	C16—C17Aa—H17A	109.5
C10—C5—C4	119.46 (15)	C19—C18—N3	120.65 (16)
C10—C5—C6	120.27 (16)	C16—C17Aa—H17B	109.5
C5—C6—H6	120.0	C19—C18—C23	120.39 (17)
C7—C6—C5	120.09 (19)	C23—C18—N3	118.92 (15)
C7—C6—H6	120.0	C16—C17Aa—H17C	109.5
C6—C7—H7	120.2	C16—C17Bb—H17D	109.5
C6—C7—C8	119.64 (19)	C18—C19—H19	120.4
C8—C7—H7	120.2	C18—C19—C20	119.14 (19)
C7—C8—H8	119.3	C16—C17Bb—H17E	109.5
C9—C8—C7	121.39 (19)	C16—C17Bb—H17F	109.5
C9—C8—H8	119.3	C20—C19—H19	120.4
C8—C9—H9	120.1	C19—C20—H20	119.6
C8—C9—C10	119.9 (2)	C21—C20—C19	120.9 (2)
C10—C9—H9	120.1	C21—C20—H20	119.6
N1—C10—C5	122.43 (15)	C20—C21—H21	120.3
N1—C10—C9	118.83 (17)	C20—C21—C22	119.37 (19)
C17Aa—C16—O15	109.5 (5)	C22—C21—H21	120.3
C5—C10—C9	118.73 (17)	C21—C22—H22	119.5
C2—S11—C12	99.06 (8)	C21—C22—C23	121.0 (2)
C17Bb—C16—O15	113.9 (5)	C23—C22—H22	119.5
S11—C12—H12A	108.8	C18—C23—C22	119.24 (19)
C17Aa—C16—H16A	109.8	C18—C23—H23	120.4
S11—C12—H12B	108.8	C22—C23—H23	120.4
C17Aa—C16—H16B	109.8		
			
N1—C2—N3—C4	−0.6 (3)	C7—C8—C9—C10	0.0 (4)
N1—C2—N3—C18	176.02 (16)	C8—C9—C10—N1	178.3 (2)
N1—C2—S11—C12	0.18 (17)	C8—C9—C10—C5	−1.0 (3)
C2—N1—C10—C5	0.7 (3)	C10—N1—C2—N3	−1.3 (3)
C2—N1—C10—C9	−178.59 (18)	C10—N1—C2—S11	178.30 (13)
C2—N3—C4—C5	2.9 (2)	C10—C5—C6—C7	−1.1 (3)
C2—N3—C4—O23	−176.56 (16)	S11—C2—N3—C4	179.77 (12)
C2—N3—C18—C19	97.9 (2)	S11—C2—N3—C18	−3.6 (2)
C2—N3—C18—C23	−84.4 (2)	S11—C12—C13—O14	−19.1 (3)
C2—S11—C12—C13	−68.94 (15)	S11—C12—C13—O15	162.70 (14)
N3—C2—S11—C12	179.82 (13)	C12—C13—O15—C16	176.3 (3)
N3—C4—C5—C6	176.06 (15)	O14—C13—O15—C16	−1.9 (4)
N3—C4—C5—C10	−3.3 (2)	C13—O15—C16—C17Bb	161.5 (9)
N3—C18—C19—C20	177.27 (18)	C13—O15—C16—C17Aa	97.7 (7)
N3—C18—C23—C22	−177.5 (2)	C18—N3—C4—C5	−173.88 (14)
C4—N3—C18—C19	−85.4 (2)	C18—N3—C4—O23	6.7 (2)
C4—N3—C18—C23	92.4 (2)	C18—C19—C20—C21	−0.4 (4)
C4—C5—C6—C7	179.52 (18)	C19—C18—C23—C22	0.3 (4)
C4—C5—C10—N1	1.7 (3)	C19—C20—C21—C22	1.5 (4)
C4—C5—C10—C9	−179.01 (18)	C20—C21—C22—C23	−1.7 (5)
C5—C6—C7—C8	0.0 (3)	C21—C22—C23—C18	0.8 (5)
C6—C5—C10—N1	−177.70 (16)	O23—C4—C5—C6	−4.5 (3)
C6—C5—C10—C9	1.6 (3)	O23—C4—C5—C10	176.07 (17)
C6—C7—C8—C9	0.6 (4)	C23—C18—C19—C20	−0.5 (3)

### Hydrogen-bond geometry (Å, º)

**Table:**

*D*—H···*A*	*D*—H	H···*A*	*D*···*A*	*D*—H···*A*
C7—H7···O23^i^	0.93	2.59	3.452 (3)	155
C12—H12*B*···O14^ii^	0.97	2.42	3.311 (3)	153
C19—H19···O23^ii^	0.93	2.41	3.236 (2)	148

Symmetry codes: (i) −*x*+3/2, *y*−1/2, −*z*+1/2; (ii) *x*, *y*+1, *z*.

## References

[bb1] Alanazi, A. M., Abdel-Aziz, A. A.-M., Shawer, T. Z., Ayyad, R. R., Al-Obaid, A. M., Al-Agamy, M. H. M., Maarouf, A. R. & El-Azab, A. S. (2016). *J. Enzyme Inhib. Med. Chem.* **31**, 721–735. 10.3109/14756366.2015.1060482 26162029

[bb2] Al-Khuzaie, M. G. A. & Al-Majidi, S. M. H. (2014). *Iraqi J. Sci.* **55**, 582–593.

[bb3] Al-Majidi, S. M. H. & Al-Khuzaie, M. G. A. (2015). *Asian J. Chem.* **27**, 756–762.

[bb4] Al-Suwaidan, I. A., Abdel-Aziz, A. A. M., Shawer, T. Z., Ayyad, R. R., Alanazi, A. M., El-Morsy, A. M., Mohamed, M. A., Abdel-Aziz, A. I., El-Sayed, M. A. A. & El-Azab, A. S. (2016). *J. Enzyme Inhib. Med. Chem.* **31**, 78–89. 10.3109/14756366.2015.1004059 25815668

[bb5] Al-Suwaidan, I. A., Abdel-Aziz, A. A. M., Shawer, T. Z., Ayyad, R. R., Alanazi, A. M., El-Morsy, A. M., Mohamed, M. A., Abdel-Aziz, A. I., El-Sayed, M. A. A. & El-Azab, A. S. (2017). *J. Enzyme Inhib. Med. Chem.* **32**, 1229–1239. 10.3109/14756366.2015.1004059 25815668

[bb6] Deshmukh, M. B. & Dhongade, S. (2004). *E-J. Chem.* **1**, 17–31.

[bb7] Dolomanov, O. V., Bourhis, L. J., Gildea, R. J., Howard, J. A. K. & Puschmann, H. (2009). *J. Appl. Cryst.* **42**, 339–341.

[bb8] El-Azab, A. S., Abdel-Hamide, S. G., Sayed-Ahmed, M. M., Hassan, G. S., El-Hadiyah, T. M., Al-Shabanah, O. A., Al-Deeb, O. A. & El-Subbagh, H. I. (2013). *Med. Chem. Res.* **22**, 2815–2827.

[bb9] Elfekki, I. M., Hassan, W. F. M., Elshihawy, H. E. A. E., Ali, I. A. I. & Eltamany, E. H. M. (2014). *Chem. Pharm. Bull.* **62**, 675–694. 10.1248/cpb.c14-00158 24990505

[bb10] El-Helby, A. G. A. & Wahab, M. H. A. (2003). *Acta Pharm.* **53**, 127–138. 14764247

[bb11] El-Sayed, S., Metwally, K., El-Shanawani, A. A., Abdel-Aziz, L. M., Pratsinis, H. & Kletsas, D. (2017). *Chem. Cent. J.* **11**, 102–111. 10.1186/s13065-017-0333-x PMC5640562 29086906

[bb12] Gawad, N. M. A., Georgey, H. H., Youssef, R. M. & El-Sayed, N. A. (2010). *Eur. J. Med. Chem.* **45**, 6058–6067. 10.1016/j.ejmech.2010.10.008 21051122

[bb13] Godhani, D. R., Jogel, A. A., Sanghani, A. M. & Mehta, J. P. (2016). *Indian J. Chem.* **55B**, 734–746.

[bb14] Groom, C. R., Bruno, I. J., Lightfoot, M. P. & Ward, S. C. (2016). *Acta Cryst.* B**72**, 171–179. 10.1107/S2052520616003954 PMC4822653 27048719

[bb15] Gursoy, A. & Karal, N. (2003). *Eur. J. Med. Chem.* **38**, 633–643. 10.1016/s0223-5234(03)00085-0 12832136

[bb16] Khalil, A. A., Hamide, S. G. A., Al-Obaid, A. M. & El-Subbagh, H. I. (2003). *Arch. Pharm. Med. Chem.* **2**, 95–103. 10.1002/ardp.200390011 12761762

[bb17] Lfta, S. J., Ayram, N. B. & Baqer, S. M. (2016). *Al-Nahrain J. Sci.* **19**, 1–12.

[bb18] Lv, X., Yang, L., Fan, Z. & Bao, X. (2018). *J. Saudi Chem. Soc.* **22**, 101–109.

[bb19] Mohamed, M. A., Ayyad, R. R., Shawer, T. Z., Abdel-Aziz, A. A. M. & El-Azab, A. S. (2016). *Eur. J. Med. Chem.* **112**, 106–113. 10.1016/j.ejmech.2016.02.002 26890117

[bb20] Nguyen, C. T., Nguyen, Q. T., Dao, P. H., Nguyen, T. L., Nguyen, P. T. & Nguyen, H. H. (2019). *J. Chem.*, Article ID 1492316, 8 pp (https://doi. org/10.1155/2019/1492316)

[bb21] Pandey, S. K., Singh, A. & Nizamuddin, A. S. (2009). *Eur. J. Med. Chem.* **44**, 1188–1197. 10.1016/j.ejmech.2008.05.033 18614258

[bb22] Rigaku OD (2018). *CrysAlis PRO*. Rigaku Oxford Diffraction, Yarnton, UK.

[bb23] Rimaz, M., Khalafy, J., Tavana, K., Slepokura, K., Lis, T., Souldozi, A., Mahyari, A. T., Shajari, N. & Ramazani, A. (2009). *Z. Naturforsch. Teil B*, **64**, 1065–1069.

[bb24] Saeed, A., Mahmood, S. & Florke, U. (2014). *Turk. J. Chem.* **38**, 275–287.

[bb25] Sheldrick, G. M. (2015*a*). *Acta Cryst.* A**71**, 3–8.

[bb26] Sheldrick, G. M. (2015*b*). *Acta Cryst.* C**71**, 3–8.

[bb27] Turner, M. J., McKinnon, J. J., Wolff, S. K., Grimwood, D. J., Spackman, P. R., Jayatilaka, D. & Spackman, M. A. (2017). *CrystalExplorer17.* University of Western Australia. http://hirshfeldsurface.net

